# Super-enhancer in prostate cancer: transcriptional disorders and therapeutic targets

**DOI:** 10.1038/s41698-020-00137-0

**Published:** 2020-11-19

**Authors:** Xuanrong Chen, Qianwang Ma, Zhiqun Shang, Yuanjie Niu

**Affiliations:** Department of Urology, Tianjin Institute of Urology, The Second Hospital of Tianjin Medical University, 300211 Tianjin, China

**Keywords:** Prostate cancer, Cancer epigenetics

## Abstract

Abnormal activity of oncogenic and tumor-suppressor signaling pathways contributes to cancer and cancer risk in humans. Transcriptional dysregulation of these pathways is commonly associated with tumorigenesis and the development of cancer. Genetic and epigenetic alterations may mediate dysregulated transcriptional activity. One of the most important epigenetic alternations is the non-coding regulatory element, which includes both enhancers and super-enhancers (SEs). SEs, characterized as large clusters of enhancers with aberrant high levels of transcription factor binding, have been considered as key drivers of gene expression in controlling and maintaining cancer cell identity. In cancer cells, oncogenes acquire SEs and the cancer phenotype relies on these abnormal transcription programs driven by SEs, which leads to cancer cells often becoming addicted to the SEs-related transcription programs, including prostate cancer. Here, we summarize recent findings of SEs and SEs-related gene regulation in prostate cancer and review the potential pharmacological inhibitors in basic research and clinical trials.

The hallmarks of cancer, including sustaining proliferation, activating invasion, metastasis, and aberrant replicative immortality, are closely connected to cancer-specific regulatory mechanisms of gene expression^1^. Prostate cancer (PCa) is one of the leading causes of cancer-related deaths in men worldwide^2,3^. Although clinically beneficial treatment options for localized PCa include surgery, radiotherapy, and androgen ablation therapy, there is basically no cure for metastatic castration-resistant PCa (CRPC)^4–6^. Therefore, it is most necessary than ever to further understand the crucial regulators within the PCa and develop new therapies^7^.

The enhancer is a class of regulatory DNA sequence that defines the genetic regulatory circuitry^8^. It increases the promoter activity to activate target genes through specific transcription factors (TF)^9^. For cancer-type-specific gene expression, enhancer closely interact with the promoter, over either short or long distances, independent of the corresponding orientation and position concerning the transcription start sites (TSS)^10,11^. Enhancers often contain conserved recognition DNA sites for RNA polymerase, TFs, and co-activators and function as binding platforms^10–12^. Accumulated evidence reveals that enhancers are bound by epigenetic modifications, such as mono-methylation at H3 lysine 4 (H3K4me1) and acetylation at H3 lysine 27(H3K27ac), generally^13,14^. In addition, the three-dimensional structure of enhancers to TSS affects the interaction activity and expression output, with the distance varying from less than 10 Kb to more than 1 Mb^15,16^.

Super-enhancer (SE) is defined as large clusters of enhancers spanning across a long-range region of genomic DNA that drives stronger transcriptional activation ability than individual enhancers^4^. Similar to the typic enhancer, SE incorporates modelized regulation mechanisms. Specific TFs bind to SE to trigger promoter-enhancer interaction, mediated by chromatin looping, to load the SE to the cognate promoter. Then the basal machinery is recruited to initiate the downstream transcription activity (depicted in Fig. 1). The SE was first proposed in mouse embryonic stem cells (mESCs), by chromatin immunoprecipitation (ChIP)-sequencing analysis of active histone marker (H3K27ac) and other TFs^17^. The ROSE (Rank ordering of super-enhancers) algorithm is designed to search SEs by locating genomic proximity for grouping elements to a putative target gene^17,18^. Generally accepted models of SEs are considered to be large clusters of regulatory elements (after over 20 Kb) binding with dense transcriptional coactivators, such as BRD4 and CDK7, and with high potential to activate target gene expression output^19^ (summarized in Table 1). As shown in mESCs, pluripotency genes like OCT4, SOX2, and NANOG are all controlled and activated by SEs, given the concept that SEs can drive specific gene expression that controls and defines the cell identity and engages in cell-type-specific biological processes^20^. It is worth noting that the expression level of SE-related genes is significantly higher than that of the typical enhancer-control genes, which has been widely validated in a variety of cancer types^21^. On the other hand, this addiction makes SEs and SE-related genes as potential therapeutic targets and diagnosis^22^.

**Fig. 1:**
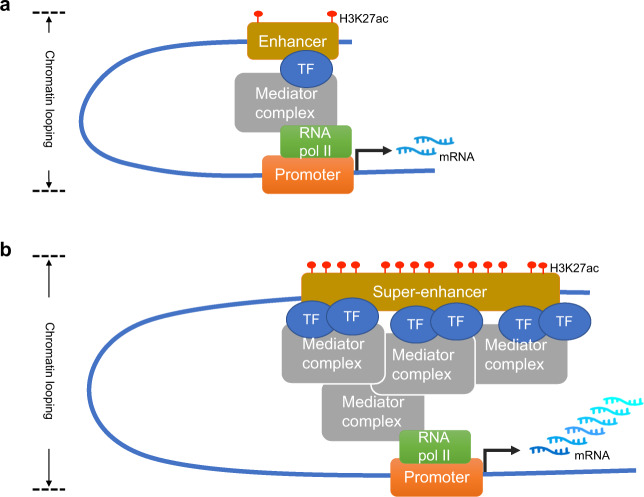
The structure and function of enhancer and super-enhancer. **a** Schematic structure of the typical enhancer. **b** Schematic structure of the super-enhancer. H3K27ac, acetylation of histone 3 lysine 27; TF, transcription factor; RNA pol II, RNA polymerase II; mRNA, messenger RNA.

**Table 1:** The main difference between enhancer and super-enhancer.

Regulatory element	H3K4me1 mark	H3K4me3 mark	H3K27ac mark	TF binding	Mediator binding	Transcriptional output
Enhancer	+	–	+	+	+	+/++
Super-enhancer	+	–	+++	+++	+++	+++

*H3K4me1* H3 lysine 4 monomethylation, *H3K4me3* H3 lysine 4 trimethylation, *H3K27ac* H3 lysine 27 acetylation, *TF* transcription factor.

“+” and “–” indicate the presence or absence, respectively.

The most cutting-edge research has revealed that membrane-less organelles, such as the nucleolus, stress granule, processing body, and nuclear speckles form subcellular compartments to facilitate signaling transduction and transcriptional regulation by liquid–liquid phase separation^23–25^. In essence, the SE can be described as a co-assembly of high-density transcription factors, co-factors, chromatin regulators, non-coding RNA, and RNA polymerases^26,27^. Computer simulations and experimental validations indicate that, in the context of the number and valence of interacting components and the affinity of the interaction between transcription factors and nucleic acids, phase separations play essential roles in the SE assembly and function^26,28^. Inhibition of the activity of SE function highlights sensitivity and vulnerability in cancers along with cancer-specific oncogene downregulation, which could be a possible therapeutic target for cancer treatment^29^. SE complexes are dynamic, highly enriched condensates, particularly sensitive to SE-related inhibition^26,28^. The essential components of SE are dysregulated in PCa, such as increased MED1 phosphorylation at T1457 in a CDK7-dependent manner in metastatic CRPC and enzalutamide-resistant cells. Bromodomain-containing proteins (BRDs) are overexpressed in CRPC and show particular prognostic value in PCa.

Given the cancer-specific SE complexes in PCa, we summarize the critical transcriptional regulation and therapeutic targets of the SEs and SE-associated regulator proteins and provide promising future directions for SEs in the prostate cancer community (depicted in Fig. 2).

**Fig. 2:**
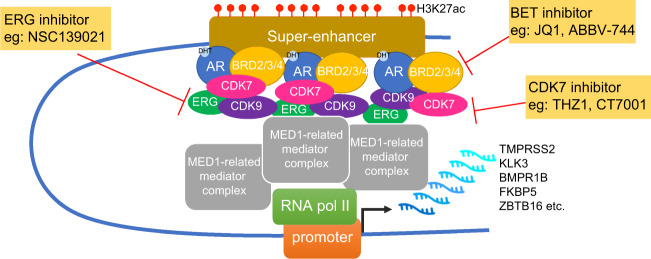
Schematic illustration of super-enhancer-mediated action in prostate cancer. Proposed mode of the mechanism of super-enhancer-mediated action in prostate cancer. H3K27ac, acetylation of histone 3 lysine 27; AR, androgen receptor; BRD2/3/4, bromodomain containing protein 2/3/4; CDK7, cyclin dependent kinase 7; CDK9, cyclin dependent kinase 9; ERG, erythroblast transformation-specific-related gene; MED1, mediator complex subunit 1; RNA pol II, RNA polymerase II.

## SE-related protein BET/BRD4 in prostate cancer

The bromodomain and extra-terminal domain (BET) family proteins act as “readers” of acetylated histones, and are important transcriptional regulators. BRD2, BRD3, BRD4, and BRDT (bromodomain testis-specific protein) are part of the BET family^30^. BRDT is most commonly expressed in germ cells, while the other three are ubiquitously expressed. BRD2, BRD3, BRD4, and BRDT share an extra-terminal domain and conserved N-terminal bromodomains (BD1 and BD2). BRD4, as a chromatin reader, recognizes acetylated histones and facilitates the transcription activation by propelling the recruitment of the positive transcription elongation factor P-TEFB^31^. In particular, BRD4 shows a density binding activity in SE and drives the cell-identical gene expression^32^. In PCa, AR-positive or AR-signaling-component cell lines (VCaP, LNCaP, and 22RV1) are selectively sensitive to BRD4 inhibition, but not in AR-negative cell lines (PC3 and DU145)^33,34^. BRD4 has been found that sequence-specific DNA-binding TFs may physically interact with it in a gene-specific manner, such as AR^35^. Mechanically, BRD4 physically interacts with the N-terminal domain of AR, and BRD4 colocalizes with AR at AR target loci to drive AR-mediated gene transcription. For the BMPR1B gene, AR-and BRD4-associated binding signals in the enhancers and SEs are significantly induced by dihydrotestosterone (DHT) treatment. The expression levels of BMPR1B are corroborating with the ChIP-seq data. Moreover, almost half of the known BRD-containing proteins are related to PCa, which also contribute the main chromatin-related processes and changes in PCa, beyond BRD4^36^. These BRD-containing proteins (such as ATAD2, BRD8, CREBBP, and KTM2A) perform a wide range of downstream functions by recognizing acetylated histones, also present to be TFs, AR co-activators or methyltransferases^37–39^. Therefore, BRD-containing proteins are important transcriptional regulators that initiate chromatin restructuring beyond the SE function.

AR signaling remains the most common resistance mechanism in most CRPC patients^40^. On one hand, BRD4, by functioning downstream of AR signaling, appears to be effectively blocking the oncogenic drivers of PCa and less likely to be bypassed by acquired treatment resistance of AR therapy. BRD4 inhibition preferentially blocks both BRD4 and AR recruitment (the BRD4 and AR cistrome) to target loci on a genome-wide scale and leads to defects in transcriptional elongation. The acquisition of BRD4-associated SEs leads to prompt expression of key oncogenic genes, including TMPRSS2-ETS, KLK3, and BMPR1B in PCa, especially CRPC. Urbanucci et al. demonstrated that the transcription of certain BET proteins (BRD2 and BRD4), and ATAD2, a recently reported common coactivator of AR, may be induced by androgen^41^. The upregulated AR leads to a positive loop that boosts the expression of BRDs to expand the AR cistrome by increasing chromatin accessibility. On the other hand, BET inhibitors can also operate via mechanisms other than AR signaling. Shah et al. demonstrated that BET inhibitors resensitized drug-resistant tumors to enzalutamide by inhibiting the glucocorticoid receptor^42^. Cai et al. showed that the BRD4 inhibitor, JQ1, significantly inhibited androgen-independent growth of CRPC cells in vitro and in vivo by blocking AR-V7 chromatin binding and transcriptional programs co-activated by AR-V7 and ZFX in addition to the canonical AR signaling^43^.

### SE-related protein CDK7 in prostate cancer

The mammalian cyclin-dependent kinases (CDKs) contain subfamilies with specific functions related to cell cycle (CDK1, CDK2, CDK4, CDK6) and transcriptional regulation (CDK7, CDK8, CDK9, CDK12, and CDK19)^44,45^. CDK7 is a ubiquitously expressed kinase that activates the cell cycle controlling through phosphorylation of CDKs and plays a key role in transcription regulation^46^. The mRNA and protein expression levels of CDK7 alone showed no significant change in benign cells and PCa cells^47^. However, CDK7 activity affects chromatin modification by promoting the recruitment of histone methyltransferases SETD1A/B and SETD2, which are phosphorylated by the C-terminal domain^48,49^. CDK7 also phosphorylates a range of TFs, including p53 and nuclear hormone receptors (RAR, AR, ER, etc.), to promote the transcription activation activity and subsequent protein-degradation^50–53^. Studies show that CDK7 inhibition can transiently block the functional activity and expression of the oncogenic drivers (AR, ETS, MYC, and E2F) in prostate cancer, and all these changes require the involvement of MED1 as a cofactor^47,54–56^. CDK7 activates MED1 via ligand-specific phosphorylation, and the IDRs (intrinsically disordered region) of MED1 form phase-separation properties to be recruited to the AR-bound SEs, resulting in the high-density assembly of the SE-related transcription apparatus. CDK7 inhibition has been effectively tested in several aggressive cancer types, including MYCN-amplified neuroblastoma, T-cell acute lymphoblastic leukemia, triple-negative breast cancer, and small-cell lung cancer^50,57–60^. The CDK7-related SEs mediate the recruitment of AR and RNA polymerase II to boost the expression of a host of target genes, known as the “Achilles cluster” genes, thus becoming transcriptional addictive and sensitive to CDK7 inhibition^50^.

### SE-related protein ERG in prostate cancer

The erythroblast transformation-specific (ETS) family proteins are the essential transcription factors necessary for cell-type-specific lineage differentiation and expression patterns^61^. ERG protein is the master transcription factor for endothelial, hematopoietic, and luminal cell differentiation^62–65^. It interacts with other transcription factors by forming complexes to establish the cell-type-specific patterns. In PCa, ERG expression and rearrangement is per se not a strong prognostic biomarker and only relevant in the context of a specific molecular subtype^66–68^. Gerke et al. found that it was crucial to determine the prognostic value with other biomarkers, such as RRM2 and TYMS, when applying the ERG status to predict the outcomes^69^. ChIP-seq data show ERG binds to the vast majority of SEs in VCaP (a TMPRSS2-EGR fusion-positive cell line) cell^65^. The SE-associated lineage-specific machinery of ERG is linked with the TFs like FOXA1 and HOXB13, which play important roles in prostate-cancer-specific gene expression. Mechanically, ERG increases the SEs activity partially through the BRG1-associated chromatin remodeling complex to establish accessible chromatin of SEs and transcriptional regulation. In summary, ERG drives the prostate-cancer-specific lineage genes by regulating SEs. TMPRSS2-ERG structural rearrangements occur in close to 50% of PCa patients and contribute to the ERG overexpression^70^. The proposed model of ERG overexpression was previously thought to be driven by TMPRSS2 promoter hijacking. Ken et al. showed that TMPRSS2, along with the rearranged ERG allele, formed an expanded SE^71^. The expanded SE still contains cis-regulatory elements and extends into the ERG locus, which promotes ERG overexpression. This work firstly confirms that the expansion of the SE region after chromosomal rearrangements could positively drive the target gene expression. As for ERG, it synergistically regulates by physically interacting with prostate-cancer-specific regulators AR, HOXB13, and FOXA1. The regulatory landscape difference between TMPRSS2-ERG fusion and non-fusion PCa types may depend on the SE-related ERG-specific transcriptional profile, including activated NOTCH pathway. In light of the present findings, we conclude that ERG contributes to the SE-driven oncogenic transcriptional addiction, and that SEs lead to the overexpression of the ERG gene, leading to subsequent overexpression of ERG-target genes that drive the development of PCa.

### Other SE-associate factors in prostate cancer

Abundant evidence shows that the transcriptional regulatory regions of SEs are practically enriched with cancer-related single nucleotide polymorphisms (SNPs), resulting in dysregulation of target genes and contribution to cancer development^72–74^. Chen et al. demonstrated that high enrichment of PCa-specific risk variants in SE regions, particularly in the disease-specific regulatory regions and the DNA regulatory elements, may lead to prostate carcinogenesis^75^. O-GlcNAc transferase (OGT), a glycosyltransferase, catalyzes the addition of a single O-GlcNAc sugar to serine and threonine residues^76^. OGT, as a major metabolic integration point in human cells, is also involved in the hexosamine biosynthetic pathway (HBP) to increase O-GlcNAcylation-modified nuclear proteins^77^. RNA polymerase II is reported to be the most prominent O-GlcNAcylation-modified protein that regulates the formation of the transcriptional pre-initiation complex formation. Also, increased OGT expression has been found in many cancers, including prostate cancer, where high O-GlcNAc protein levels are associated with poor clinical prognosis. Harri et al. showed that OGT regulated SE-dependent transcription through chromatin compaction^78^. Over 70% of the SE-related genes are related to the OGT chromatin mark, and the OGT inhibition significantly decreases the SE-related mRNA expression. The activity of OGT is required for the expression of MYC-target mitotic proteins. Thus, OGT could be an effective target for MYC-addicted PCa.

AR is a predominant target for PCa, owing to the functional AR signaling in the early-and late-stage PCa. Simon et al. demonstrated that R1881-activated AR bound at a comparable number of SE sites in VCaP cells, and indicated that AR attributed to the SE-associated action^79^. The androgen-regulated SE-target genes are associated with cell-proliferation-associated gene sets, stem cell properties, and hallmark AR signaling, such as KLK2, KLK3, TMPRRSS2, and FKBP5. In addition, abundant evidence shows that AR also tightly cross-talks with other factors^80^. In low androgen environments or AR-overexpression status, AR promotes the expression of some SE-complex-associated proteins, such as BRD4^41^. AR closely interacts with the BRDs in the N-terminal domain (NTD) instead of the ligand-binding domain (LBD), since the NTD was essential for the transcriptional activity of AR^81^. Alternative splicing variants of the AR that lack LBD are overexpressed in patients who are resistant to enzalutamide or the androgen synthesis inhibitor abiraterone acetate^82^. For AR mutants (full-length AR with point-mutated forms and AR with lack of LBD splice variants and nonsense mutants), such as AR-V7, AR T878A, and AR H875Y, some BET inhibitors (PFI-1 and BETi) could reduce these mutants’ expression by regulating RNA processing and reducing alternative splicing^83,84^. In this regard, we suspect that the formation of AR-bound-SE may be partly affected and the cistrome of AR mutants could redistribute while retaining some binding sites. The current inhibitors may still be effective for AR mutants, but the extent of the effect still needs to be specifically evaluated.

The forkhead box A1 (FOXA1) transcription factor plays a pivotal role for the development and differentiation of several endoderm-derived organs, including prostate^85^. FOXA1 directly binds to and de-compacts condensed chromatin to increase accessibility of the binding sites for partnering nuclear hormone receptors, including estrogen receptor and AR^86^. Lupien et el. showed that FOXA1 functionally collaborated with AR and was predominantly recruited to the AR-regulated enhancers and SEs to establish a lineage-specific program in PCa^87^. FOXA1 plays a key role in prostate tumorigenesis by reprogramming the AR cistrome to new binding sites and driving the transformation of normal prostate epithelial cells^88^. Besides, FOXA1 also interacts directly with AR^89^. Considering the AR-bound-SE complexes, the alterations in FOXA1 may impact subsequent effects on the AR cistrome and lineage-specific programs in PCa.

### Promising pharmacological targets

SEs drive PCa cells into becoming addicted to dysregulated transcriptional programs mediated by BRD4, CDK7, ERG, and other factors, but also become a powerful rationale for therapeutic interventions. Targeting SE may disrupt the dysregulated networks of the oncogenic functions, and some small molecule inhibitors and blockers have been tested to selectively target PCa cells. We summarize these inhibitors and their mechanisms as below and an overview description in Table 2.

**Table 2:** Overview of selected inhibitors in prostate cancer.

Target	Compound	Status	Identifier
ERG	YK-4-279	Preclinical	
	NSC139021	Preclinical	
BET/BRD4	GSK525762	Phase 1 active, not recruiting	NCT03150056
	GS-5829	Phase 1 completed	NCT02607228
	ABBV-075	Phase 1 completed	NCT02391480
	ABBV-744	Phase 1, recruiting	NCT03360006
	ZEN003694	Phase 1 completed	NCT02705469
	ZEN003694	Phase 1 active, not recruiting	NCT02711956
	ZEN003694	Phase 2 active, not recruiting	NCT04471974
CDK7	THZ1	Preclinical	
	SY-1365	Phase 1 active, not recruiting	NCT03134638
	CT7001	Phase 1, recruiting	NCT03363893
	CT7001	Phase 2, recruiting	NCT03363893

Only the most advanced clinical studies are shown.

*ERG* erythroblast transformation-specific-related gene, *BET* the bromodomain and extra-terminal domain, *BRD4* bromodomain containing protein 4, *CDK7* cyclin dependent kinase 7.

First, BET proteins have been targeted by JQ1 in preclinical models in vitro and in vivo^90^. JQ1 exhibits a high binding affinity to the bromodomain pocket and displaces BRD4 from the active chromatin in most SE sites and is believed to act predominantly on BRD4, BRD2, BRD3, and, BRDT. In acute myeloid leukemia (AML) and myeloma, pre-clinical models and clinical trials already show the BRD4 inhibition induces strong suppression of tumor progression^91^. In CRPC xenograft mouse models, BRD4 inhibition (JQ1) is more effective than direct AR antagonism (enzalutamide)^33,84^. This novel approach can be used to synergistically block the oncogenic drivers in advanced PCa for better treatments. BRD4 contains two conserved bromodomains, BD1 and BD2. Dual-bromodomain BET inhibitors are designed to competitively inhibit the binding of the BD1 and BD2, such as OTX015, CPI-0610, and ABBV-075^92–96^. However, in some monotherapy clinical trials, dose-limiting adverse events, such as reduced numbers of thrombocytes in the blood and some gastrointestinal toxicity, are limited the clinical activity. Fairre et al. proposed a highly potent and selective inhibitor of the BD2 domain by a medicinal chemistry campaign named ABBV-744^97^. ABBV-744 selectively maintains high activity in PCa cell lines and xenografts and has a lower toxicity than ABBV-075. Further analyses demonstrate that ABBV-744 displaces BRD4 from AR-bound SE sites and disrupts the AR-target transcriptional programs. The successful development of the preclinical compound JQ1 for BET inhibition has also enabled several compounds of BET inhibitors (ABBV-075, ABBV-744, GSK525762, ZEN003694, and GS-5829) to successfully enter the clinical trials.

THZ1, a covalent inhibitor of CDK7, shows the ability to suppress the CDK7-dependent phosphorylation activity to achieve clinical activity^58^. The inhibition of CDK7 by THZ1 is related to the global transcriptional downregulation at high dose levels, but studies have found that cancer cell lines are sufficiently sensitive to lower doses of THZ1. Further reports indicate that THZ1 may selectively target SE-driven transcription programs, including MYC-dependent transcription amplification and the expression of other cancer-specific oncogenic TFs and signaling molecules^98,99^. Rasool et al. demonstrated that THZ1 attenuated the AR-signaling and maintained efficacy in CRPC and enzalutamide-resistant PCa cells^47^. Also, CDK7 selective inhibitors have been developed, such as SY1365 and CT7001, and evaluated in clinical trials in other advanced solid tumors^100,101^.

ERG overexpression is observed in a large group of primary PCa and CRPC. A highly selective small-molecule inhibitor of ERG, NSC139021, inhibits the growth of ERG-positive cancer cells^102^. Another small-molecule inhibitor, YK-4-279, reduces the ERG-positive PCa patient-derived xenograft growth^103^.

Advances in SE profiling and chromatin landscape profiling make it possible for identifying essential SE regulators and SE-target genes in cancers^104,105^. Also, the relevant information will feed to the pharmacologic industry for therapeutic interventions.

### Future directions

Super-enhancers are a large cluster of active transcriptional enhancers and are rich in enhancer-related chromatin features. Compared to typical enhancers, SEs are larger, exhibit a higher density of TFs, and are often associated with critical lineage-specific genes.

In PCa, fundamental components of the SE complexes, such as BDR4 and ERG, densely bind to the enhancer and SE elements to promote tumorigenesis and tumor growth. In advanced CRPC patients, the SE activity created by these transcription factors remains stable and evolves to be more specific, which makes it possible that SEs may be promising therapeutic targets^47,97,106,107^.

Several pre-clinical and clinically relevant studies targeting SEs have been successfully carried out in various tumors, including PCa^21^. BRD4 inhibition has been validated and investigated in leukemia, lymphoma, myeloma, neuroblastoma, breast cancer, prostate cancer, and other cancer types. Compared to castration-sensitive status, advanced CRPC patients exhibit an AR deregulation status^41^. Evidence shows that BRDs (BRD2, BRD4, and ATAD2) are prognostic markers that are overexpressed in CRPC. AR-deregulation-mediated BRDs upregulation intensifies the SE activity and feeds back a positive loop for AR chromatin binding. In other words, AR deregulation forms a transcription addiction status by enhancing the SE activities. To define the BET inhibition response in CRPC, a ten-gene signature, BROMO-10, was designed and used to guide patient selection for combinatorial trials of the BET inhibition against other agents. Remarkably, the BROMO-10 signature still needs to be refined and evaluated for AR signaling status and BRDs expression as well. Using this method, we can design specific gene signatures to evaluate the efficacy of other inhibitors and screen patients for potential therapeutic benefits. CDK7 inhibition has shown surprising therapeutic effects in preclinical models without evident systemic toxicity, which gives us high hopes for further human clinical trials^108^. Besides, it has been reported that SEs tend to undergo double-strand breaks and are therefore susceptible to deficiencies in cellular DNA-repair mechanisms. This shows that the combination of endocrine therapy and drugs targeting DNA damage repair will improve the anti-tumor efficiency, and clinical studies have already been conducted. Strikingly, Ma et al. found that SEs reorganization was tightly linked to drug resistance^109^. In repetitive cisplatin-treated cancer cells, the developmental transcription factor ISL1 invokes an unconventional trans-differentiation identity via SE reorganization to escape the drug-induced near-to-death status and facilitate tumor colonization. This may raise the question of how to effectively target cancer cells by avoiding the acquisition resistance in a SE-reorganization manner. The inhibition of transcription factors, such as SOX10 and ISL1, might potentially blockade the SE reorganization beyond BRDs or CDK7 inhibitors.

However, researchers have discovered that SE-related inhibitors might not have therapeutic effects in certain PCa types^110,111^. PCa cell lines and organoids from individuals with SPOP mutations show therapeutic resistance to cell growth arrest and apoptosis induced by BRD4 inhibitors^112,113^. The resistance to BRD4 inhibition in SPOP-mutant PCa can be overcome by combining with AKT inhibitors, and SPOP mutations may be used as biomarkers to guide the choice of treatment options for PCa patients, including those with urgent needs seeking precision medicine, and to determine whether the treatment is valid or not^114,115^. Moreover, it is worth noting that targeting SEs for cancer can cause side effects that cannot be ignored, because blocking SEs may also suppress specific SE-dependent tumor suppressor genes that are associated with cancer risk, such as cancer cell death^116,117^. Therefore, before using SEs as a therapeutic approach for the treatment of specific cancers, we urgently need to do more detailed research and make effective decisions.
